# Elterliches Belastungserleben, Unaufmerksamkeits‑/Hyperaktivitätssymptome und elternberichtete ADHS bei Kindern und Jugendlichen: Ergebnisse aus der KiGGS-Studie

**DOI:** 10.1007/s00103-024-03859-9

**Published:** 2024-03-27

**Authors:** Laura Neuperdt, Ann-Kristin Beyer, Stephan Junker, Elvira Mauz, Heike Hölling, Robert Schlack

**Affiliations:** Abteilung Epidemiologie und Gesundheitsmonitoring, Fachgebiet Psychische Gesundheit, Robert Koch-Institut, General-Pape-Str. 62–66, 12101 Berlin, Deutschland

**Keywords:** Aufmerksamkeitsdefizit‑/Hyperaktivitätsstörung, Finanzielle Sorgen, Erziehungsprobleme, Studie zur Gesundheit von Kindern und Jugendlichen in Deutschland (KiGGS), Deutschland, Attention deficit hyperactivity disorder, Financial worries, Parenting problems, German Health Interview and Examination Survey for Children and Adolescents (KiGGS), Germany

## Abstract

**Hintergrund:**

Eine Aufmerksamkeitsdefizit‑/Hyperaktivitätsstörung (ADHS) bei Kindern und Jugendlichen geht mit elterlichen Belastungen einher. Umgekehrt sind psychosoziale Belastungen der Eltern mit dem Auftreten von Unaufmerksamkeits‑/Hyperaktivitätssymptomen (UHS) bei den Kindern assoziiert. In diesem Beitrag wird der Zusammenhang verschiedener Arten und des Umfangs elterlicher Belastungen mit UHS und einer elternberichteten ADHS-Diagnose der Kinder analysiert.

**Methodik:**

Auf Grundlage der Daten von *n* = 4596 Teilnehmenden der KiGGS-Kohorte (Welle 2: 2014–2017) wurden in einer querschnittlichen Analyse elterliche Belastungen in Zusammenhang mit UHS sowie einer elternberichteten ADHS-Diagnose der Kinder gebracht. Berichtet werden Häufigkeiten, Mittelwerte sowie für Geschlecht, Alter, sozioökonomischen Status und Migrationshintergrund adjustierte Beta-Koeffizienten und Odds Ratios.

**Ergebnisse:**

Einzeln betrachtet waren mehr elterliche Belastungsarten mit UHS assoziiert als mit einer ADHS-Diagnose. Multivariat betrachtet erwiesen sich finanzielle Sorgen und Erziehungsprobleme/Konflikte mit den Kindern als signifikante Prädiktoren sowohl für UHS als auch für eine ADHS-Diagnose des Kindes. 4 oder mehr elterliche Belastungen gingen zudem mit einer höheren Wahrscheinlichkeit sowohl für UHS als auch für eine ADHS-Diagnose einher.

**Diskussion:**

Finanzielle Belastungen und Erziehungsprobleme stellen relevante Belastungen der Eltern von Kindern mit UHS oder ADHS-Diagnose dar. Wechselbeziehungen zwischen elterlichen Belastungen und der ADHS eines Kindes sind anzunehmen. Maßnahmen zur familiären Entlastung können entweder auf eine Verbesserung der familiären Lage (Verhältnisprävention) oder des familiären Umgangs mit dem von ADHS betroffenen Kind (Verhaltensprävention) zielen.

## Abstract (en)

**Background:**

A child’s attention deficit hyperactivity disorder (ADHD) is associated with strain for the parents. In turn, psychosocial parental strain is associated with higher probabilities for the occurrence of inattention/hyperactivity symptoms (IHS) in their children. The aim of this paper is to assess the association between parental strain, IHS, and a parent-reported ADHD diagnosis of the children.

**Methodology:**

Based on data from *n* = 4596 participants of the KiGGS cohort (wave 2: 2014–2017), the type and extent of parental strain was set in relation to IHS and an ADHD diagnosis of the child in cross-sectional analysis. Frequencies, means, beta coefficients, and odds ratios adjusted for sex, age, socioeconomic status, and migration background are reported.

**Results:**

In individual consideration, a greater number of parental strains were associated with IHS than with an ADHD diagnosis. In a multivariate analysis, financial worries and parenting problems/conflicts with the children were significant predictors of IHS and an ADHD diagnosis, respectively. In addition, four or more types of parental strain were associated with a higher likelihood of both IHS and an ADHD diagnosis.

**Discussion:**

Financial and child-rearing strain are relevant to parents of children with IHS and an ADHD diagnosis. Interrelationships between parental stress and a child’s IHS or an ADHD diagnosis can be assumed. To relieve their burden, prevention can either aim at improving the situation of ADHD-affected families or at improving the family’s handling of the child’s ADHD.

## Hintergrund

Die Aufmerksamkeitsdefizit‑/Hyperaktivitätsstörung (ADHS) ist eine der häufigsten psychischen Störungen im Kindes- und Jugendalter [1]. In Deutschland beträgt die Prävalenz einer elternberichteten ADHS-Diagnose bei Kindern und Jugendlichen 4,4 % [2]. Aufgrund der hohen Heritabilität der ADHS hat die Forschung bislang schwerpunktmäßig auf die neurobiologischen und genetischen Aspekte der ADHS fokussiert, während psychosoziale Aspekte im Kontext der ADHS erst in jüngerer Zeit verstärkte Aufmerksamkeit erhielten [3–7]. Die ADHS eines Kindes ist mit einer Vielzahl individueller und sozialer Belastungen auch für die Eltern verbunden. So geht die ADHS eines Kindes in der Familie mit einer verringerten elterlichen gesundheitsbezogenen Lebensqualität [8] sowie mehr elterlichen psychopathologischen Symptomen [9] einher. Darüber hinaus können den Eltern finanzielle Einschränkungen oder Probleme sowie Nachteile in der beruflichen Entwicklung entstehen, wenn sie z. B. aufgrund der Erkrankung des Kindes ihre Arbeitszeit reduzieren oder ganz auf eine Erwerbstätigkeit verzichten müssen. Eltern von Kindern mit ADHS berichten zudem häufiger von einer geringeren Arbeitseffizienz, von Jobkündigungen sowie weniger Arbeitsangeboten [10, 11]. Ein Kind mit ADHS in der Familie erhöht zudem die Wahrscheinlichkeit von Partnerschaftsproblemen der Eltern und ist mit höheren familiären Trennungs- und Scheidungswahrscheinlichkeiten assoziiert [11, 12].

Umgekehrt sind Stress und psychosoziale Belastungen der Eltern auch mit höheren Wahrscheinlichkeiten für das Auftreten von psychischen Problemen bei ihren Kindern verbunden [13–15]. So beschreiben Bolster et al. [13], dass Kinder und Jugendliche, deren Eltern Belastungen in den Bereichen Familie, Erziehung, Finanzen, berufliche Situation oder Partnerschaft angeben, häufiger psychisch auffällig sind als Kinder aus Familien ohne solche Belastungen [13]. Bei kumulativen elterlichen Belastungen ist die Wahrscheinlichkeit für das Vorliegen psychischer Auffälligkeiten bei Kindern noch einmal deutlich erhöht [13]. In einer Studie mit 255 Elternpaaren wurde gezeigt, dass elterlicher Stress, vermittelt über ein ungünstiges Erziehungsverhalten (z. B. zu hohe Nachsichtigkeit oder Überreagieren), und eine dysfunktionale elterliche Konfliktkommunikation mit expansivem kindlichen Problemverhalten verbunden sind, das durch oppositionelles Trotzverhalten, Störungen der Aufmerksamkeit, Hyperaktivität und Störungen des Sozialverhaltens gekennzeichnet ist [14]. Bezogen auf ADHS legen 2 aktuelle Metaanalysen nahe, dass psychosoziale elterliche Belastungen, z. B. durch Kindererziehung, Partnerschaftsprobleme, kritische Lebensereignisse, Alleinerziehung, niedrigen sozioökonomischen Status (SES), elterlichen Stress oder elterliche Psychopathologie, signifikant mit der Wahrscheinlichkeit des Vorliegens einer ADHS-Diagnose sowie der Ausprägung von ADHS-Symptomen bei den Kindern verbunden sind [16, 17]. In einer weiteren Übersichtsarbeit wird darauf hingewiesen, dass Partnerschaftsprobleme und -konflikte der Eltern zur Aufrechterhaltung und/oder Verstärkung von kindlichen ADHS-Symptomen beitragen können, die wiederum verstärkend auf die Probleme in der elterlichen Paarbeziehung zurückwirken [18].

Diese bisherigen Forschungsbefunde zu psychosozialen elterlichen Belastungsfaktoren und ADHS haben vorrangig auf die funktionellen Aspekte der Eltern-Kind-Beziehung (wie Erziehungsverhalten oder elterliche Psychopathologie), qualitative Aspekte der Partnerbeziehung zwischen den Eltern oder strukturelle Risikofaktoren wie SES oder Einelternfamilie fokussiert. Weniger bekannt ist dagegen, welcher Zusammenhang zwischen subjektiv empfundenen elterlichen Belastungen und ADHS-Symptomen beziehungsweise einer ADHS-Diagnose bei den Kindern besteht. In der vorliegenden Arbeit werden diese Zusammenhänge sowohl dimensional, unter Berücksichtigung der Unaufmerksamkeits‑/Hyperaktivitätsskala des Strengths and Difficulties Questionnaire (SDQ), als auch kategorial, mittels Einbezug einer elternberichteten ADHS-Diagnose, betrachtet. Auf diese Weise können sowohl subklinische Ausprägungen einer ADHS-Symptomatik als auch klinisch manifestierte ADHS untersucht werden.

Ziel dieser Arbeit ist es, zu einer besseren Kenntnis über mögliche Zusammenhänge eines breiten Spektrums elterlichen Belastungserlebens und des Vorliegens von Unaufmerksamkeits‑/Hyperaktivitätssymptomen (UHS) beziehungsweise ADHS-Diagnose der Kinder auf der Basis einer bundesweiten Stichprobe beizutragen. Dabei werden auch die Effekte kumulativer Belastungen betrachtet. Dabei werden folgende Hypothesen untersucht:

1. Das Vorliegen von verschiedenen, subjektiv empfundenen Belastungen der Eltern geht mit mehr UHS bzw. einer höheren Wahrscheinlichkeit des Vorliegens einer ADHS-Diagnose ihrer Kinder einher.
2. Mit steigender Anzahl elterlicher Belastungen liegen ausgeprägtere UHS beziehungsweise eine größere Häufigkeit elternberichteter ADHS-Diagnosen bei den Kindern vor.

## Methodik

### Stichprobe

Die vorliegenden Analysen beruhen auf einer querschnittlichen Auswertung der Daten von insgesamt 4596 Teilnehmenden im Alter von 11–17 Jahren der KiGGS-Kohorte zum Zeitpunkt der KiGGS-Welle 2 (2014–2017), davon 2339 Mädchen und 2257 Jungen [19]. Die KiGGS-Kohorte ist eine Längsschnittstudie im Rahmen des Gesundheitsmonitorings am Robert Koch-Institut, für die sämtliche wiederbefragungsbereite Teilnehmende der KiGGS-Basiserhebung (2003–2006; [20]) erneut befragt und in Teilen auch untersucht wurden. Die Responserate in dem Befragungsteil der Erhebung, der der vorliegenden Analyse zugrunde lag, betrug 61,5 %. Einzelheiten des Studiendesigns der KiGGS-Kohorte sind bei Mauz et al. [21] und Lange et al. [22] beschrieben.

### Instrumente

#### ADHS.

In der KiGGS-Studie wurden die Eltern gefragt, ob bei ihrem Kind jemals eine Aufmerksamkeitsdefizit‑/Hyperaktivitätsstörung festgestellt wurde. Laut KiGGS-Falldefinition liegt eine ADHS bei einem Kind dann vor, wenn die Eltern eine jemals von einer Ärztin/einem Arzt oder einer Psychologin/einem Psychologen gestellten ADHS-Diagnose berichteten [23–25].

#### Unaufmerksamkeit/Hyperaktivität.

Symptome von Hyperaktivität und Unaufmerksamkeit des Kindes (nachfolgend als Unaufmerksamkeits‑/Hyperaktivitätssymptome (UHS) bezeichnet) wurden in der KiGGS-Studie mit der Subskala „Unaufmerksamkeit/Hyperaktivität“ des SDQ im Elternbericht erhoben [26]. Die Subskala besteht aus 5 Items zu den 3 oben genannten Kernsymptomen, der Recall-Zeitraum umfasst die letzten 6 Monate. Die Fragen sind auf einer 3‑stufigen Likert-Skala („0 = trifft nicht zu“, „1 = trifft teilweise zu“ und „2 = trifft eindeutig zu“) zu beantworten. Für die dimensionale Auswertung wurde der Summenscore der Subskala mit einem Wertebereich zwischen 0 und 10 herangezogen. Höhere Werte auf der Summenskala entsprechen einer stärkeren Ausprägung der UHS.

#### Elterliche Belastungen.

Subjektiv empfundene elterliche Belastungen wurden mit der Skala „Elterliche Belastungen“ von Sperlich et al. [27] erhoben. Mit dieser Skala werden 13 verschiedene Arten elterlicher Belastungen erfragt: Belastung im Haushalt, finanzielle Sorgen, ständiges Dasein und „Einsatz“ für die Familie, alleinige Verantwortung für Kindererziehung, Belastung aufgrund pflegebedürftiger Familienangehörigen, Erziehungsprobleme/Konflikte mit den Kindern, Konflikte mit der (Ex‑)Partnerin/dem (Ex‑)Partner, Konflikte mit anderen Familienangehörigen, ungewolltes Alleinleben/Einsamkeit, berufliche Situation/Arbeitslosigkeit, geringe Anerkennung bei Haus‑/Familienarbeit, Belastung aufgrund eines chronisch kranken oder behinderten Kindes, Vereinbarkeitsproblemen von Familie und Beruf. Vorangestellt war eine Frage nach dem allgemeinen Belastungsempfinden insgesamt im Alltag. Die Stärke der Belastung konnte jeweils für die verschiedenen Belastungsarten auf einer Ratingskala von „1 = überhaupt nicht/trifft nicht zu“, „2 = ein wenig“, „3 = mittelmäßig“, „4 = stark“ bis „5 = sehr stark“ bewertet werden. Für die vorliegenden Analysen wurden für jede Belastungsart in Anlehnung an das Vorgehen von Sperlich et al. [28] und Bolster et al. [13] die Antwortoptionen 4 und 5 zu der Kategorie „belastet“ sowie 0, 1, 2 und 3 zu der Kategorie „nicht belastet“ zusammengefasst [13, 28]. Auf der Grundlage der 13 einzelnen Belastungsarten wurde zudem ein Summenscore als kumulativer Belastungsindex mit den Ausprägungen „keine“, „1“, „2“, „3“ und „4 und mehr“ Belastungen gebildet.

#### Confounder.

Da sich in den bisherigen Analysen der KiGGS-Daten erhebliche Unterschiede sowohl in Bezug auf das Vorliegen von UHS als auch hinsichtlich der Häufigkeit von elternberichteten ADHS-Diagnosen nach Alter, Geschlecht, SES, Migrationshintergrund und Familienstruktur gezeigt hatten [2, 23, 25, 29], wurden diese Variablen hier als soziodemografische Kontrollvariablen (Confounder) für die multivariaten Analysen berücksichtigt. Darüber hinaus wurden das Alter (in vollendeten Lebensjahren) und das Geschlecht („1 = männlich“; „2 = weiblich“) der Kinder und Jugendlichen als Kontrollvariablen herangezogen. Der familiäre SES wird in der KiGGS-Studie mit einem Index erfasst, der auf Angaben der Eltern zu ihrem Bildungsabschluss, ihrer beruflichen Stellung und zum Haushaltsnettoeinkommen beruht und in 3 Kategorien (Statusgruppen) „1 = niedrig“ (unterstes Quintil), „2 = mittel“ (2. bis 4. Quintil) und „3 = hoch“ (5. Quintil) eingeteilt ist [30, 31]. Bezüglich des Migrationshintergrunds wurde unterschieden, ob dieser nicht, über einen oder über beide Elternteile vorliegt („0 = ohne“; „1 = einseitig“; „2 = beidseitig“). Ein beidseitiger Migrationshintergrund liegt definitionsgemäß dann vor, wenn entweder mindestens ein Elternteil und das Kind selbst aus einem anderen Land zugewandert sind oder wenn das Kind in Deutschland geboren wurde, aber beide Elternteile aus einem anderen Land in die Bundesrepublik zugewandert sind oder nicht die deutsche Staatsangehörigkeit besitzen. Ein einseitiger Migrationshintergrund bedeutet, dass nur ein Elternteil nicht die deutsche Staatsangehörigkeit besitzt oder aus einem anderen Land zugewandert ist [32]. Bezüglich der Familienstruktur wurden 4 Kategorien „1 = Kernfamilie mit leiblichen Eltern“; „2 = Stieffamilie“; „3 = Eineltern-Familie“ und „4 = Nicht bei Eltern lebend“ gebildet.

### Statistische Analysen

Die statistische Signifikanz der Zusammenhänge von UHS sowie dem Vorliegen einer elternberichteten ADHS-Diagnose bei den Kindern und Jugendlichen mit unterschiedlichen elterlichen Belastungsarten wurde für die UHS mithilfe nicht adjustierter linearer Regressionsanalysen, für das Vorliegen einer ADHS-Diagnose mit dem Rao-Scott-Chi-Square-Test überprüft. Mittelwerte und Häufigkeiten wurden mit 95 %-Konfidenzintervallen (KI) getestet.

Um die Effekte einzelner Belastungsarten sowie kumulativer elterlicher Belastungen unter Kontrolle der oben genannten Confounder auf UHS und auf das Vorliegen einer elternberichteten ADHS-Diagnose einerseits sowie unter zusätzlicher wechselseitiger Kontrolle aller erhobenen Belastungsarten andererseits zu untersuchen, wurden darüber hinaus lineare Regressionsanalysen mit der SDQ-Hyperaktivitätsskala und binäre logistische Regressionsanalysen mit dem Vorliegen einer elternberichten ADHS-Diagnose (Ja/Nein) als abhängige Variablen durchgeführt. Die Modellierung erfolgte dabei in 2 Schritten: Zunächst wurde für jede einzelne elterliche Belastungsart je ein Modell mit Adjustierung durch die genannten Kontrollvariablen berechnet. In einem zweiten Schritt wurden sämtliche Belastungsarten (ohne „Belastungen im Allgemeinen“) in einem Gesamtmodell sowohl für UHS als auch für das Vorliegen einer ADHS-Diagnose berücksichtigt. Darüber hinaus wurde je ein separates Modell für kumulative Belastungen, einmal für UHS und einmal für die elternberichtete ADHS-Diagnose als abhängige Variable gerechnet. Bei den Modellen mit UHS als abhängige Variable wurden Kinder und Jugendliche mit ADHS-Diagnose ausgeschlossen, um die Gruppen besser trennen zu können.

Die Datenanalyse erfolgte mit der Statistiksoftware Stata (Version 17). Für sämtliche Analysen wurde die komplexe Survey-Struktur der KiGGS-Daten durch die Verwendung der svy-Prozedur berücksichtigt. Mittels einer Gewichtung wurde die Kohortenstichprobe an die Ausgangsstichprobe zum Zeitpunkt der KiGGS-Basiserhebung angepasst [22]. Hierfür wurde die Nichtteilnahmewahrscheinlichkeit unter Berücksichtigung relevanter soziodemografischer und gesundheitsbezogener Variablen mittels eines logistischen Regressionsmodells berechnet und die Gruppen mit einer niedrigeren Wiederteilnahmebereitschaft anschließend stärker gewichtet. Für eine ausführliche Beschreibung des Gewichtungsprozederes siehe Lange et al. [22].

## Ergebnisse

Tab. 1 zeigt die deskriptiven Statistiken der KiGGS-Teilstichprobe, die diesen Analysen zugrunde liegt. Mädchen und Jungen waren annähernd gleich vertreten (48,5 % Mädchen). 40,9 % der Kinder und Jugendlichen waren in der Altersgruppe von 11–13 Jahren und 59,1 % waren 14–17 Jahre alt. Der Skalenmittelwert für UHS lag bei M = 2,69, die Häufigkeit einer elternberichteten ADHS-Diagnose bei 6,6 %. Die Häufigkeit einzelner elterlicher Belastungen reichte von 2,2–19,5 %. Allgemein belastet zu sein, gaben 27,0 % der befragten Eltern an.

**Table 1:** 

	*n* (ungewichtet)	Anteil in % mit 95 %-KI (gewichtet)
*Geschlecht*		
Männlich	2257	51,5 (50,2–52,9)
Weiblich	2339	48,5 (47,1–49,8)
*Alter*		
11–13 J	1973	40,9 (39,5–42,3)
14–17 J	2623	59,1 (57,7–60,5)
*SES*		
Niedrig	523	18,6 (16,6–20,9)
Mittel	2987	63,4 (61,1–65,7)
Hoch	994	17,9 (16,0–20,1)
*Migrationshintergrund*		
Kein	3742	71,8 (68,4–75,0)
Einseitig	360	9,8 (8,5–11,3)
Beidseitig	464	18,4 (15,7–21,4)
*Familienstruktur*		
Kernfamilie mit leiblichen Eltern	3570	77,6 (75,5–79,6)
Stieffamilie	388	8,6 (7,4–9,8)
Eineltern-Familie	541	13,1 (11,6–14,7)
Nicht bei Eltern lebend	29	0,8 (0,5–1,4)
**Unaufmerksamkeits‑/Hyperaktivitätssymptome (Unaufmerksamkeits‑/Hyperaktivitätsskala SDQ)** Mittelwert (95 %-KI): 2,69 (2,60–2,79)	4596	–
*ADHS-Diagnose*		
Ja	262	6,6 (5,6–7,6)
Nein	4231	93,4 (92,4–94,4)
*Anzahl elterlicher Belastungen*	4220	–
0	2197	51,2 (47,1–53,2)
1	757	17,0 (15,5–18,6)
2	449	11,3 (10,1–12,6)
3	315	7,7 (6,8–8,8)
4 und mehr	501	12,9 (11,5–14,4)
*Art der Belastung (Antwortkategorie „belastet“)*		
Belastung allgemein	1250	27,0 (25,2–28,8)
Belastung im Haushalt	797	17,7 (16,3–19,2)
Finanzielle Sorgen	545	14,6 (13,1–16,2)
Ständiges Dasein und „Einsatz“ für die Familie	857	19,5 (18,0–21,0)
Alleinige Verantwortung für Kindererziehung	495	12,5 (11,2–14,1)
Pflegebedürftige Familienangehörige	280	6,2 (5,3–7,2)
Erziehungsprobleme/Konflikte mit den Kindern	434	10,6 (9,5–11,9)
Konflikte mit der (Ex‑)Partnerin/dem (Ex‑)Partner	366	8,6 (7,5–9,8)
Konflikte mit anderen Familienangehörigen	231	5,6 (4,7–6,7)
Ungewolltes Alleinleben/Einsamkeit	131	3,7 (3,0–4,7)
Berufliche Situation/Arbeitslosigkeit	508	12,3 (10,8–13,9)
Geringe Anerkennung Haus‑/Familienarbeit	421	10,7 (9,5–12,1)
Chronisch krankes oder behindertes Kind	103	2,2 (1,8–2,8)
Vereinbarkeitsprobleme von Familie und Beruf	470	9,9 (8,8–11,1)

### Elterliche Belastungen und UHS von Kindern und Jugendlichen

Tab. 2 zeigt die Ergebnisse der Zusammenhangsanalysen zwischen elterlichen Belastungen und UHS von Kindern und Jugendlichen. Signifikant höhere Werte auf der Hyperaktivitätsskala des SDQ wurden bei höherer Belastung der Eltern in 7 von 13 Belastungsbereichen (finanzielle Sorgen, alleinige Verantwortung für die Kindererziehung, Erziehungsprobleme/Konflikte mit den Kindern, Konflikte mit der (Ex‑)Partnerin/dem (Ex‑)Partner, ungewolltes Alleinleben/Einsamkeit, berufliche Situation/Arbeitslosigkeit sowie geringe Anerkennung der Haus‑/Familienarbeit) gefunden. Dies traf auch auf Kinder von Eltern zu, die angaben, sich im Allgemeinen belastet zu fühlen. Kein Zusammenhang bestand mit den Bereichen Belastung im Haushalt, ständiges Dasein und „Einsatz“ für die Familie, pflegebedürftige Familienangehörige, Konflikte mit anderen Familienangehörigen, chronisch krankes oder behindertes Kind, Vereinbarkeitsprobleme von Familie und Beruf.

**Table 2:** 

Art der Belastung	*n*	Eltern ohne Belastungen MW (95 %-KI)	Eltern mit Belastungen MW (95 %-KI)	*p*-Wert
Belastungen allgemein	4100	2,44 (2,35–2,54)	2,65 (2,50–2,81)	0,024
Belastung im Haushalt	4126	2,47 (2,38–2,55)	2,67 (2,45–2,89)	0,083
Finanzielle Sorgen	4115	2,41 (2,32–2,50)	3,09 (2,80–3,37)	< 0,001
Ständiges Dasein und „Einsatz“ für die Familie	4116	2,48 (2,40–2,57)	2,60 (2,40–2,81)	0,311
Alleinige Verantwortung für Kindererziehung	4115	2,44 (2,36–2,53)	2,92 (2,66–3,18)	< 0,001
Pflegebedürftige Familienangehörige	4113	2,50 (2,41–2,58)	2,49 (2,23–2,74)	0,936
Erziehungsprobleme/Konflikte mit den Kindern	4135	2,38 (2,30–2,47)	3,52 (3,21–3,83)	< 0,001
Konflikte mit der (Ex‑)Partnerin/dem (Ex‑)Partner	4121	2,44 (2,36–2,53)	3,03 (2,72–3,34)	< 0,001
Konflikte mit anderen Familienangehörigen	4125	2,48 (2,39–2,57)	2,72 (2,40–3,05)	0,152
Ungewolltes Alleinleben/Einsamkeit	4111	2,47 (2,38–2,56)	3,17 (2,72–3,63)	0,004
Berufliche Situation/Arbeitslosigkeit	4123	2,44 (2,35–2,53)	2,83 (2,60–3,06)	0,002
Geringe Anerkennung Haus‑/Familienarbeit	4108	2,44 (2,35–2,53)	3,01 (2,74–3,27)	< 0,001
Chronisch krankes oder behindertes Kind	4116	2,49 (2,41–2,58)	2,55 (1,94–3,16)	0,844
Vereinbarkeitsprobleme von Familie und Beruf	4113	2,47 (2,38–2,56)	2,66 (2,44–2,88)	0,101
*Anzahl der Belastungen*	3929	–	–	–
Keine	2080	2,30 (2,18–2,41)	–	Ref.
1	693	–	2,57 (2,41–2,73)	0,005
2	416	–	2,72 (2,43–3,01)	0,007
3	293	–	2,60 (2,28–2,92)	0,077
4 und mehr	447	–	3,01 (2,77–3,24)	< 0,001

Die Signifikanz der Gruppenunterschiede wurde mit univariater linearer Regressionsanalyse getestet; *Ref.* Referenzkategorie

Tab. 3 zeigt die Ergebnisse der multivariaten linearen Regressionsanalysen für jede Belastungsart mit UHS der Kinder als abhängige Variable (Modell 1). Nach Adjustierung für Geschlecht, Alter, SES, Migrationshintergrund und Familienstruktur blieben in folgenden Bereichen die signifikanten Zusammenhänge zwischen elterlichen Belastungsarten mit UHS bei den Kindern bestehen: finanzielle Sorgen (*p* < 0,001), alleinige Verantwortung für die Kindererziehung (*p* = 0,004), Erziehungsprobleme/Konflikte mit den Kindern (*p* < 0,001), Konflikte mit der (Ex‑)Partnerin/dem (Ex‑)Partner (*p* < 0,001), ungewolltes Alleinleben/Einsamkeit (*p* = 0,008), berufliche Situation/Arbeitslosigkeit (*p* = 0,006) sowie geringe Anerkennung der Haus‑/Familienarbeit (*p* < 0,001). Mit Bezug auf kumulative Belastungen war das Vorliegen von 1 (*p* = 0,001), 2 (*p* = 0,008) und 4 und mehr (*p* < 0,001) Belastungsarten bei den Eltern mit mehr UHS der Kinder assoziiert. Bei Einbezug aller Belastungsarten in einem volladjustierten Gesamtmodell (Modell 2) wurde ersichtlich, dass nur noch folgende Belastungsarten mit signifikant mehr Hyperaktivität verbunden waren: „finanzielle Sorgen“ (*p* = 0,005) und „Erziehungsprobleme/Konflikte mit den Kindern“ (*p* < 0,001).

**Table 3:** 

	Modell 1: Arten elterlicher Belastungen einzeln	Modell 2: Einbezug aller Belastungsarten
*Art der Belastung*	*Eltern mit Belastungen* *Beta (95* *%-KI; n* *=* *4089)*	*Eltern mit Belastungen* *Beta (95* *%-KI; n* *=* *3859)*
Belastungen allgemein	0,20 (0,02–0,37)*	–
Belastung im Haushalt	0,19 (−0,04–0,41)	−0,09 (−0,29–0,11)
Finanzielle Sorgen	0,59 (0,30–0,89)***	0,41 (0,13–0,70)**
Ständiges Dasein und „Einsatz“ für die Familie	0,09 (−0,12–0,31)	−0,22 (−0,46–0,01)
Alleinige Verantwortung für Kindererziehung	0,38 (0,12–0,64)**	0,18 (−0,08–0,44)
Pflegebedürftige Familienangehörige	−0,02 (−0,27–0,22)	−0,15 (−0,41–0,11)
Erziehungsprobleme/Konflikte mit den Kindern	1,06 (0,77–1,35)***	0,94 (0,62–1,27)***
Konflikte mit der (Ex‑)Partnerin/dem (Ex‑)Partner	0,55 (0,24–0,85)***	0,12 (−0,20–0,45)
Konflikte mit anderen Familienangehörigen	0,23 (−0,12–0,58)	−0,04 (−0,39–0,32)
Ungewolltes Alleinleben/Einsamkeit	0,64 (0,17–1,11)**	0,31 (−0,15–0,77)
Berufliche Situation/Arbeitslosigkeit	0,33 (0,10–0,57)**	0,01 (−0,23–0,25)
Geringe Anerkennung Haus‑/Familienarbeit	0,55 (0,27–0,82)***	0,23 (−0,04–0,49)
Chronisch krankes oder behindertes Kind	−0,07 (−0,65–0,50)	−0,10 (−0,77–0,58)
Vereinbarkeitsprobleme von Familie und Beruf	0,21 (−0,04–0,46)	−0,03 (−0,29–0,23)
*Anzahl der Belastungen (n* *=* *3893)*		
1	0,30 (0,12–0,47)**	–
2	0,39 (0,10–0,68)**	–
3	0,29 (−0,04–0,62)	–
4 und mehr	0,69 (0,43–0,95)***	–

Adjustiert für Geschlecht, Alter, SES, Migrationshintergrund und Familienstruktur; *p*-Werte: * *p* < 0,05 = signifikant, ** *p* < 0,01 = sehr signifikant, *** *p* < 0,001 = hochsignifikant

### Elterliche Belastungen und elternberichtete ADHS-Diagnose von Kindern und Jugendlichen

Tab. 4 zeigt die Häufigkeiten elternberichteter ADHS-Diagnosen ihrer Kinder im Zusammenhang mit elterlichen Belastungen. Signifikant häufiger wurde eine elternberichtete ADHS-Diagnose bei höherer Belastung der Eltern in 6 von 13 Belastungsbereichen (finanzielle Sorgen, ständiges Dasein und „Einsatz“ für die Familie, alleinige Verantwortung für die Kindererziehung, durch Erziehungsprobleme/Konflikte mit den Kindern, Konflikte mit der (Ex‑)Partnerin/dem (Ex‑)Partner sowie geringe Anerkennung der Haus‑/Familienarbeit) gefunden. Dies traf auch zu, wenn die Eltern angaben, im Allgemeinen belastet zu sein. Kein Zusammenhang bestand mit den Bereichen Belastung im Haushalt, pflegebedürftige Familienangehörige, Konflikte mit anderen Familienangehörigen, ungewolltes Alleinleben/Einsamkeit, chronisch krankes oder behindertes Kind, Vereinbarkeitsprobleme von Familie und Beruf.

**Table 4:** 

Art der Belastung	*n*	Eltern ohne Belastungen in % (95 %-KI)	Eltern mit Belastungen in % (95 %-KI)	*p*-Wert
Belastungen allgemein	4373	5,9 (4,9–7,2)	8,7 (6,8–11,2)	0,0184
Belastung im Haushalt	4401	6,6 (5,6–7,8)	7,0 (4,6–10,5)	0,7914
Finanzielle Sorgen	4389	5,8 (4,9–6,9)	11,6 (8,3–15,9)	0,0003
Ständiges Dasein und „Einsatz“ für die Familie	4386	5,9 (4,9–7,1)	9,3 (6,7–12,7)	0,0224
Alleinige Verantwortung für Kindererziehung	4389	6,1 (5,1–7,3)	10,5 (6,8–15,7)	0,0277
Pflegebedürftige Familienangehörige	4386	6,6 (5,6–7,7)	8,2 (4,4–15,0)	0,4894
Erziehungsprobleme/Konflikte mit den Kindern	4409	5,8 (4,8–6,9)	14,0 (9,9–19,5)	< 0,001
Konflikte mit der (Ex‑)Partnerin/dem (Ex‑)Partner	4396	6,3 (5,3–7,4)	10,9 (7,3–15,8)	0,0133
Konflikte mit anderen Familienangehörigen	4399	6,5 (5,6–7,6)	8,5 (4,0–17,3)	0,5028
Ungewolltes Alleinleben/Einsamkeit	4384	6,5 (5,5–7,6)	10,2 (3,8–24,4)	0,3585
Berufliche Situation/Arbeitslosigkeit	4395	6,3 (5,4–7,5)	8,6 (5,5–13,1)	0,2022
Geringe Anerkennung Haus‑/Familienarbeit	4378	6,1 (5,1–7,2)	10,8 (7,2–16,0)	0,0101
Chronisch krankes oder behindertes Kind	4388	6,6 (5,6–7,7)	7,3 (3,2–15,4)	0,8259
Vereinbarkeitsprobleme von Familie und Beruf	4386	6,3 (5,4–7,5)	9,3 (6,3–13,6)	0,0758
*Anzahl der Belastungen*	4219	–	–	0,0293
Keine	2197	5,3 (4,1–6,9)	–	–
1	757	–	7,8 (5,5–11,0)	–
2	449	–	6,1 (3,7–9,8)	–
3	315	–	6,4 (3,5–11,4)	–
4 und mehr	501	–	10,7 (7,3–15,5)	–

Die Signifikanz der Gruppenunterschiede wurde mit Chi^2^-Tests nach Pearson getestet

Tab. 5 zeigt die Ergebnisse der adjustierten binären logistischen Regressionsanalysen für jede Belastungsart mit dem Vorliegen der elternberichteten ADHS-Diagnose als abhängige Variable (Modell 1). Nach Adjustierung für Geschlecht, Alter, SES, Migrationshintergrund und Familienstruktur bestand eine höhere Wahrscheinlichkeit für das Vorliegen einer elternberichteten ADHS-Diagnose nur für Kinder und Jugendliche, deren Eltern finanzielle Sorgen (*p* = 0,011) berichteten, sich von ständigem Einsatz für die Familie (*p* = 0,024), den Erziehungsproblemen/Konflikten mit den Kindern (*p* = 0,007) sowie durch geringere Anerkennung von Haus- und Familienarbeit (*p* = 0,041) belastet fühlten. Mit Blick auf kumulative Belastungen lag eine signifikant höhere Wahrscheinlichkeit für eine ADHS-Diagnose bei den Kindern lediglich dann vor, wenn die Eltern 4 oder mehr Belastungen berichteten (*p* = 0,042). Unter simultaner Berücksichtigung sämtlicher Belastungsarten in einem volladjustierten Gesamtmodell verfehlten finanzielle Sorgen nur knapp die Signifikanzgrenze (*p* = 0,055), während Erziehungsprobleme/Konflikte mit den Kindern signifikant (*p* = 0,048) mit einer höheren Wahrscheinlichkeit einer ADHS-Diagnose assoziiert waren.

**Table 5:** 

	Modell 1: Arten elterlicher Belastungen einzeln (*n* = 4357)	Modell 2: Einbezug aller Belastungsarten (*n* = 4107)
*Art der Belastung*	*Eltern mit Belastungen* *OR (95* *%-KI)*	*Eltern mit Belastungen* *OR (95* *%-KI)*
Belastungen allgemein	1,42 (0,99–2,04)	–
Belastung im Haushalt	1,13 (0,71–1,79)	0,76 (0,43–1,35)
Finanzielle Sorgen	1,77 (1,14–2,74)*	1,61 (0,99–2,63)
Ständiges Dasein und „Einsatz“ für die Familie	1,63 (1,07–2,49)*	1,41 (0,81–2,47)
Alleinige Verantwortung für Kindererziehung	1,41 (0,81–2,44)	0,98 (0,52–1,83)
Pflegebedürftige Familienangehörige	1,34 (0,65–2,75)	1,13 (0,51–2,52)
Erziehungsprobleme/Konflikte mit den Kindern	2,00 (1,21–3,30)**	1,69 (1,01–2,83)*
Konflikte mit der (Ex‑)Partnerin/dem (Ex‑)Partner	1,44 (0,86–2,42)	1,04 (0,53–2,06)
Konflikte mit anderen Familienangehörigen	1,27 (0,58–2,77)	1,07 (0,51–2,23)
Ungewolltes Alleinleben/Einsamkeit	1,04 (0,34–3,20)	0,75 (0,27–2,07)
Berufliche Situation/Arbeitslosigkeit	1,11 (0,68–1,83)	0,78 (0,49–1,23)
Geringe Anerkennung Haus‑/Familienarbeit	1,67 (1,02–2,73)*	1,37 (0,83–2,27)
Chronisch krankes oder behindertes Kind	0,87 (0,32–2,33)	0,75 (0,24–2,30)
Vereinbarkeitsprobleme von Familie und Beruf	1,44 (0,87–2,38)	1,00 (0,53–1,91)
*Anzahl der Belastungen (n* *=* *4144)*		
Keine	Ref.	–
1	1,53 (0,95–2,46)	–
2	1,12 (0,64–1,98)	–
3	1,20 (0,58–2,47)	–
4 und mehr	1,74 (1,02–2,95)*	–

*Ref.* Referenzkategorie; adjustiert für Geschlecht, Alter, SES, Migrationshintergrund und Familienstruktur; *p*-Werte: * *p* < 0,05 = signifikant, ** *p* < 0,01 = sehr signifikant, *** *p* < 0,001 = hochsignifikant

## Diskussion

Ziel des vorliegenden Beitrags war es, die Beziehungen zwischen elterlichen Belastungen und UHS bzw. dem Vorliegen einer elternberichteten ADHS-Diagnose bei Kindern und Jugendlichen in einem querschnittlichen Analysedesign auf Basis der Daten der KiGGS-Kohorte zum Erhebungszeitpunkt der KiGGS-Welle 2 (2014–2017) zu untersuchen. Hierfür wurden sowohl alle 13 erhobenen elterlichen Belastungsarten einzeln als auch kumulativ mit UHS bzw. einer elternberichteten ADHS-Diagnose des Kindes in Beziehung gesetzt.

Hypothesenkonform waren verschiedene subjektiv empfundene Belastungen mit mehr UHS und einer höheren Wahrscheinlichkeit für eine ADHS-Diagnose verbunden (Hypothese 1). Im Ergebnis der nur für die Confounder adjustierten multivariaten Analyse war ein deutlich breiteres Spektrum von elterlichen Belastungen mit mehr UHS bei den Kindern (unter Ausschluss von elternberichteten Diagnosen) assoziiert als mit einer elternberichteten ADHS-Diagnose. So waren insgesamt 8 Belastungsarten signifikant mit mehr UHS der Kinder verbunden: allgemeine Belastungen, finanzielle Sorgen, alleinige Verantwortung für die Kindererziehung, Erziehungsprobleme/Konflikte mit dem Kind, ungewolltes Alleinleben/Einsamkeit, berufliche Situation/Arbeitslosigkeit, Konflikte mit der (Ex‑)Partnerin/dem (Ex‑)Partner sowie geringe Anerkennung von Haus- und Familienarbeit. Dass elterliche Belastungen mit UHS von Kindern und Jugendlichen verbunden sind, entspricht der Erwartung und auch den Ergebnissen aktueller Übersichtsarbeiten und Metaanalysen [16, 18] zu den Zusammenhängen von elterlichen Belastungen in den Bereichen Erziehung, Partnerschaft der Eltern und Familienstruktur (Eineltern-Familie) mit einer ausgeprägteren Symptomschwere bzw. der Aufrechterhaltung von ADHS-Symptomen (zu denen u. a. Hyperaktivität zählt) bei Kindern. Umgekehrt erhöhen elterliche Belastungen wie Trennung und Scheidung der Eltern das Risiko für ADHS-Symptome bei Kindern [7]. Allerdings zeigte der Befund der nur für die Confounder adjustierten multivariaten Analyse, dass die elternberichtete ADHS-Diagnose mit insgesamt nur 4 und damit nur mit halb so vielen Belastungsarten im Vergleich zu den UHS assoziiert war: finanzielle Sorgen, ständiger Einsatz für die Familie, Erziehungsprobleme/Konflikt mit den Kindern sowie geringe Anerkennung von Haus‑/Familienarbeit. Dazu anzumerken ist, dass die leitliniengerechte Diagnostik einer ADHS aufwändig ist. Neben psychodiagnostischen Interviews, ADHS-spezifischen Fragebögen, kognitiven Leistungs- und Aufmerksamkeitstests werden in der Regel auch Informationen bei weiteren Personen eingeholt, zum Beispiel bei Erzieherinnen und Erziehern oder Lehrerinnen und Lehrern (sog. Multi-Informant-Prinzip), um die Symptomatik in unterschiedlichen Lebensbereichen zu erfassen und sie bei der klinischen Diagnosestellung mit zu berücksichtigen [33]. Es muss somit eine gewisse „diagnostische Schwelle“ überschritten und die Diagnose den Eltern auch mitgeteilt werden, damit diese sie berichten können. Daher könnte vermutet werden, dass Kinder und Jugendliche mit elternberichteter Diagnose auch eine schwere Ausprägung von ADHS-Symptomen haben und die Eltern infolgedessen auch mehr Belastungserleben berichten würden, was sich in den hier dargelegten Ergebnissen allerdings nicht zeigte. Möglicherweise sind jedoch der Versorgungsgrad und damit auch die Therapie bei diagnostizierten Fällen besser, was implizierte, dass elterliche Belastungen, die mit der ADHS eines Kindes verbunden sind, mit besserer Versorgung beziehungsweise Behandlung geringer wären. In KiGGS Welle 1 gaben jedoch 57,2 % und in KiGGS Welle 2 insgesamt 61,7 % der Eltern von Kindern mit elternberichteter ADHS-Diagnose an, weder eine pharmakologische noch eine psychotherapeutische Behandlung erhalten zu haben [2].

Im Gesamtmodell (wechselseitig adjustiert für sämtliche elterliche Belastungsarten und alle einbezogenen Confounder) waren jedoch nur noch die Belastungsarten „finanzielle Sorgen“ sowie „Erziehungsprobleme/Konflikte mit den Kindern“ signifikant sowohl mit mehr UHS als auch mit einer elternberichteten ADHS-Diagnose der Kinder assoziiert. Diese Ergebnisse verdeutlichen die finanziellen und familiären Belastungen, die mit der ADHS eines Kindes verbunden sind, etwa weil die berufliche Entwicklung eines oder beider Elternteile aufgrund des chronischen Charakters der Krankheit eingeschränkt ist und die ADHS des Kindes die familiären Beziehungen belastet [10, 11].

Kinder und Jugendliche, deren Eltern mehr als eine Belastung berichteten, wiesen hypothesenkonform auch mehr UHS auf (Hypothese 2). Bei Vorliegen von 4 und mehr Arten elterlicher Belastungen war auch die Wahrscheinlichkeit erhöht, dass das Kind eine elternberichtete ADHS-Diagnose aufwies. Ähnliche Ergebnisse zeigten die Analysen von Bolster et al. bezüglich psychischer Auffälligkeiten der Kinder: Dort waren psychische Auffälligkeiten bei Kindern und Jugendlichen bei Vorliegen mehrerer elterlicher Belastungsarten ebenfalls häufiger [13]. Auch wenn sich aus den hier berichteten Ergebnissen keine lineare Dosis-Wirkungs-Beziehung ableiten lässt, verdeutlichen sie doch, dass mit zunehmender Zahl elterlicher Belastungen auch die Wahrscheinlichkeit von mehr UHS bzw. dem Vorliegen einer elternberichteten ADHS-Diagnose bei den Kindern besteht.

### Limitationen

Für den vorliegenden Beitrag wurden die zum Zeitpunkt von KiGGS Welle 2 erhobenen Daten der KiGGS-Kohorte querschnittlich ausgewertet. Die Stichprobe wurde dabei mittels einer Gewichtungsprozedur an die Ausgangsstichprobe der KiGGS-Basiserhebung angepasst [34]. Die Daten sind daher nicht notwendigerweise für die Kinder- und Jugendbevölkerung zum Erhebungszeitpunkt 2014–2017 repräsentativ. Die Querschnittsanalysen lassen zudem keine kausalen Aussagen zu. Für die Untersuchung von kausalen Fragestellungen sind Längsschnittanalysen mit mehreren Messzeitpunkten erforderlich.

Es ist wahrscheinlich, dass sich elterliche Belastungen aufgrund der dadurch bedingten verminderten elterlichen Resilienz auf die Wahrnehmung des kindlichen Verhaltens durch die Eltern auswirkt. Es ist daher nicht auszuschließen, dass belastete Eltern das Verhalten ihrer Kinder als „auffälliger“ beurteilen als nicht belastete. Für diesbezügliche mögliche Verzerrungen kann mit diesem Studienansatz nicht kontrolliert werden. Sowohl die Erhebung der UHS als auch der ADHS-Diagnose der Kinder und Jugendlichen basieren auf einer Elternauskunft. Zwar wurde bezüglich der ADHS-Diagnose erfragt, ob diese ärztlich oder psychologisch, also von medizinischem Fachpersonal, gestellt wurde, dies lässt jedoch keine Rückschlüsse darauf zu, ob die Diagnostik leitliniengerecht erfolgte [2]. Die Selbstangaben zu den unterschiedlichen Belastungen stellen ebenfalls eine subjektive Einschätzung der Eltern dar. Die UHS des SDQ aus 5 Items [26] bilden zudem eine ADHS-Symptomatik nicht vollumfassend ab. Allerdings wurde versucht, diesem Mangel dadurch approximativ Abhilfe zu schaffen, dass die elternberichtete ärztlich oder psychologisch gestellte ADHS-Diagnose berücksichtig wurde.

Darüber hinaus ist anzumerken, dass ADHS ätiologisch vorrangig auf genetische Faktoren zurückgeführt wird [1, 3, 35], weshalb in multivariaten Analysen eine bestehende elterliche Psychopathologie, insbesondere auch eine mögliche elterliche ADHS, als Kontrollvariable berücksichtigt werden sollte [17]. Sofern die Eltern selbst von ADHS betroffen sind, ist mit Beeinträchtigungen ihrer psychischen Gesundheit [36], dem Vorliegen eines niedrigeren Bildungsniveaus [36, 37], einer höheren Wahrscheinlichkeit für Arbeitslosigkeit [36], mehr Partnerschafts- und Familienkonflikten [35, 36] sowie einem potenziell beeinträchtigten Erziehungsverhalten gegenüber den Kindern [38] zu rechnen. Diese Faktoren können sich wiederum ungünstig auf die Kinder auswirken und zu einer Verstärkung von ADHS-Symptomen beitragen [7]. Information zu elterlicher Psychopathologie beziehungsweise zu elterlicher ADHS liegen im Datensatz der KiGGS-Kohorte jedoch nicht vor.

## Fazit und Ausblick

Der vorliegende Beitrag zeigt Zusammenhänge zwischen elterlichen Belastungen und UHS bzw. einer elternberichteten ADHS-Diagnose bei Kindern und Jugendlichen in Deutschland auf. Nach den Ergebnissen dieser Untersuchung sind insbesondere finanzielle Belastungen sowie Belastungen durch die Kindererziehung mit mehr UHS bzw. einer höheren Wahrscheinlichkeit einer ADHS-Diagnose bei den Kindern assoziiert. Zudem ist wahrscheinlich, dass Wechselbeziehungen zwischen elterlichen Belastungen und UHS bzw. einer ADHS bei den Kindern und Jugendlichen bestehen [18].

Elterliche Belastungen stellen als modifizierbare umfeldbedingte Faktoren wichtige Ansatzpunkte für die Prävention und Gesundheitsförderung betroffener Familien und Kinder dar. Unterstützungsmaßnahmen können entweder auf eine Verbesserung der Situation ADHS-betroffener Familien zielen oder auf eine Verbesserung des familiären Umgangs mit dem erkrankten Kind. Verhältnispräventive Ansätze können zum Beispiel auf kommunaler Ebene die Bereitstellung familiengeeigneten Wohnraums, welcher sozial nachhaltig ist, und eines qualitativ hochwertigen Lebensumfelds mit ausreichend Spiel- und Grünflächen sein [2], auf (vor‑)schulischer Ebene die Qualifizierung von Fach‑, Lehr- und Betreuungspersonal, welche z. B. erwünschtes Verhalten der Kinder fördern und Unterstützung bei der Kompetenzentwicklung und bei Herausforderungen aufgrund der ADHS bieten können [2, 39], was zumindest mittelbar zu einer Reduktion des elterlichen Belastungserlebens beitragen könnte. Auf familiärer Ebene können die Stärkung der elterlichen Gesundheitskompetenz, Elternberatungen und -trainings zum Umgang mit kindlichem Problemverhalten oder aufsuchende Familienhilfen, die Unterstützung bei Erziehungsaufgaben, Alltagsproblemen und -konflikten bieten, einen Beitrag zur Reduktion elterlicher Belastungen im Bereich Erziehung/Konflikte mit den Kindern in ADHS-betroffenen Familien leisten [2, 40]. Solche Unterstützungsmaßnahmen sollten wissenschaftlich begleitet werden und deren Effekte hinsichtlich der Reduktion elterlichen Belastungserlebens möglichst mittels longitudinaler Datenerhebungen evaluiert werden.
